# Antigenic site of nucleoprotein gene from Indonesian rabies virus isolates

**DOI:** 10.14202/vetworld.2019.724-728

**Published:** 2019-05-31

**Authors:** Jola Rahmahani, Suwarno Suwarno, Wiwik Misaco Yuniarti, Fedik Abdul Rantam

**Affiliations:** 1 Laboratory of Virology and Immunology, Department of Veterinary Microbiology Universitas Airlangga, Jl. Mulyorejo, Kampus C Unair, Surabaya, 60111, Indonesia; 2 Department of Clinical Science, University of Airlangga, Jl. Mulyorejo, Kampus C Unair, Surabaya, 60111, Indonesia; 3 Stem Cell Research and Development Center, Universitas Airlangga, Surabaya, East Java, 60111, Indonesia

**Keywords:** amino acid, antigenic site, N gene, rabies virus

## Abstract

**Background and Aim:**

Several molecular studies on rabies virus (RABV) have been conducted in Indonesia, but it does not give clear information about molecular characteristics of previous RABV isolate in Indonesia. This study was conducted to know the characteristic of circulating RABV to determine a suitable method to control the spreading of RABV in Indonesia.

**Materials and Methods:**

Samples of infected RABV from dog brain were collected from Sumatera, Kalimantan, Sulawesi, and Bali Islands. All samples were examined based on nucleoprotein encoding gene to determine the molecular characteristics based on homology and phylogenetic tree compared to Pasteur Virus and RABV that came from another country within Asia (Indonesia, China, Thailand, India, and Korea). The collected samples were processed by one-step reverse transcriptase-polymerase chain reaction using nucleoprotein encoding gene followed by sequencing. The amino acid of its antigenic site of isolated RABV was also analyzed.

**Results:**

The results showed that isolated RABV has 84-85% similarity compared to Pasteur. According to phylogenetic construction, isolated samples do not share the same lineage toward Pasteur. The homology scores of isolated samples compared to RABV within Asia such as Indonesia, China, Thailand, India, and Korea were 98-99%, 92-93%, 88-89%, 86-88%, and 85-88%, respectively. According to antigenic site analysis compared to Pasteur, it was found that there were amino acid mutations within antigenic Site IV of nucleoprotein. Amino acid mutation from isoleucine to valine occurred in amino acid number 240 of 6 Kalimantan, 7 Kalimantan, and 8 Kalimantan. Amino acid mutation from alanine to aspartate and asparagine to threonine occurred within the same antigenic site in amino acid number 246 and 273 of C4 isolate from Sulawesi.

**Conclusion:**

According to homology and phylogenetic tree analyses, isolated RABV remained different compared to RABV within Asia and Pasteur. The amino acid mutation occurred in antigenic site of nucleoprotein encoding gene.

## Introduction

Rabies is an encephalitis disease that is caused by virus belongs to single-stranded RNA virus within *Lyssavirus* of the *Rhabdoviridae* family [1,2]. Rabies is a zoonotic disease that is transmitted through open wound or bitting of an infected animal. The human deaths due to rabies are estimated in 55,000 people/year, which is 56% of deaths in Asia and 44% in Africa. Due to high risk of the disease, the United States of America has the policy to resolve it, spending 300 million US dollars [3]. Despite being entirely preventable, human rabies is estimated to cause 59,000 global deaths annually, of which 59% occur in the Asian region [4].

Phylogenetic analysis of isolated rabies virus (RABV) in Asia using gene encoding nucleoprotein (N) sequence showed that RABV is divided into five genogroups, distributed in the Middle East, South Asia, Southeast Asia, Indonesia, and the Arctic region. Genetic relationship of this virus is the basis for developing new vaccines to control rabies in Asia [4]. Rabies genome is arranged by the nucleoprotein (N), which plays a role in encapsidation and protects the RNA from endogenous ribonuclease activity and also plays a role in transcription and replication of the virus. Phosphoprotein (P) and polymerase protein (L) are the constituent components associated with ribonucleic protein (RNP). Glycoprotein (G) is a spike-like virus-forming protein (approximately 400 thorns) and has a major role in viral attachment to the cell surface, pathogenicity, and neurovirulence of RABV. The M protein is associated with the envelope and the RNP, and may be the central protein of rhabdo-virus assembly[5,6].

N protein has a special group of antigenic determinants possessed by all RABVs. It has been recently shown to consist of four antigenic sites. Antigenic Site I and IV are constructed by linear epitopes, whereas antigenic Sites II and III are constructed by conformation-dependent epitopes [7,8]. Several molecular studies on RABV in Indonesia have been conducted, but the studies did not determine the molecular characteristics of isolated RABV in Indonesia.

To understand it, samples from the infected dogs were examined by reverse transcriptase-polymerase chain reaction (RT-PCR) using nucleoprotein encoding gene. It was followed by sequencing. Molecular analyses were conducted to determine their characteristics according to homology and phylogenetic and antigenic site analyses.

## Materials and Methods

### Ethical approval

The entire research was conducted appropriately following the ethics in using experimental animals and has been approved by the ethics commission of the Faculty of Veterinary Medicine, Universitas Airlangga, Indonesia.

### Experimental procedures

Brain specimens used in this study were collected from Sumatra (coded as 533, 391, and 438), Sulawesi (coded as C9, C3, and C4), Kalimantan (coded as 6, 7, and 8), and Bali (coded as 148, 285, and 382). Part of these samples was stored at −20°C for virus identification by RT-PCR and nucleotide sequencing. The negative control sample was obtained from an uninfected dog brain, and positive control was taken from confirmed brains of dogs infected with RABV.

The samples were subjected to RNA isolation [8]. The isolated RNA products were then processed for amplification using RT-PCR. The primers used in this process is presented in Table-1. [7]. The product of RT-PCR was visualized in 1% agarose gel electrophoresis. The gel was observed under ultraviolet 302 nm [8,9].

**Table-1:** Sequence of primer to amplify nucleoprotein encoding gene of RABV [9].

Primer	Nucleotide Sequences (5’- 3’)	Position	Sense	Primer position
RVN1F	ATGGATGCCGACAAGATTGTATTC	71-94	+	1-24
RVN1R	GAATTCCTCTCCCAGATAGCC	1097-1118	−	1027-1047

RABV=Rabies virus

Purification of DNA was done using a clean up and gel extraction kit(Nucleospin^®^ Extract II Column). Sequencing was done with Sequencer Engine AB Applied Biosystems 3130 Genetic Analyzer HITACHI automated using BigDye X terminator TM Solution kit and SAM TM Solution (Applied Biosystems). The N gene in this study was subjected to homology search using Basic Local Alignment Search Tool of NCBI (http://www.ncbi.nlm.nih.gov). The phylogenetic tree was constructed by N-J branched chain method. A tree was inferred by Bootstrap phylogenetic inference using MEGA5.05. The phylogenetic distance was observed by scoring [10].In this study, the level of virus homology of isolated viruses was compared to Indonesia, China, Thailand, and Korea RABVs submitted in GenBank. The isolated samples were also compared to Pasteur RABV.

## Results

**Figure-1:**
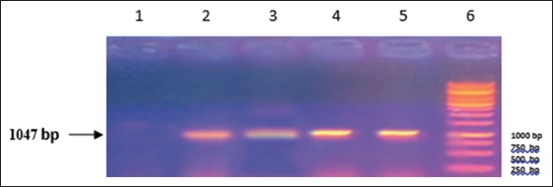
Result of amplification of rabies virus gene fragment from Sumatera (1047 bp) Column 1, negative control; 2, positive control; 3, sample code 533 from Sumatera; 4, sample code 391 from Sumatera; 5, sample code 438 from Sumatera; 6, DNA marker.

**Figure-2:**
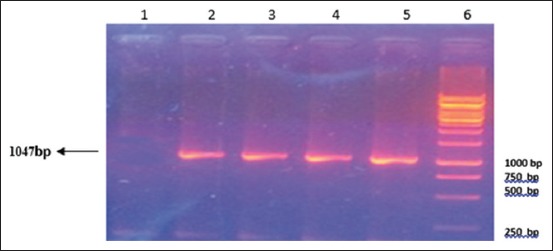
Result of amplification of rabies virus gene fragment from Sulawesi (1047 bp). Column 1, Negative control; 2, positive control; 3, sample C9 code from Sulawesi; 4, sample C3 code from Sulawesi; 5, sample C4 code from Sulawesi; 6, DNA Marker.

**Figure-3:**
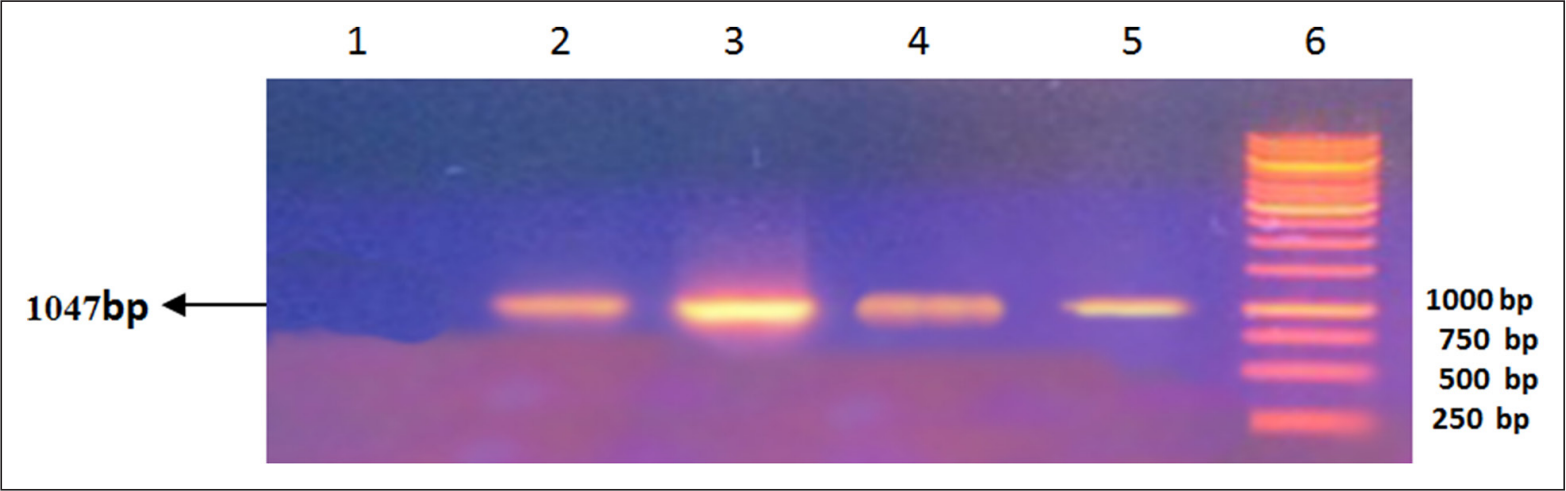
Result of amplification of rabies virus gene fragment from Kalimantan (1047 bp). Column 1, Negative control; 2, positive control; 3, sample code 6 from Kalimantan; 4, sample code 7 from Kalimantan; 5, code sample 8 from Kalimantan; 6, DNA marker.

**Figure-4:**
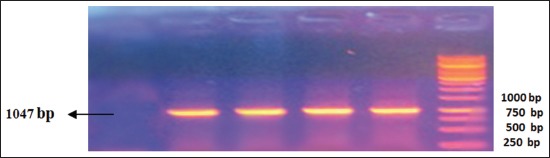
Result of amplification of rabies virus gene fragment from Bali (1047 bp). Column 1, negative control; 2, positive control; 3, sample code 148 from Bali; 4, code sample 285 from Bali; 5, sample code 382 from Bali; 6, DNA marker.

RT-PCR was performed according to nucleoprotein encoding gene. Analysis of the PCR products obtained from the amplification reaction of isolated RNA by agarose gel electrophoresis revealed the positive amplification of RABV nucleoprotein with the correct size (1047 bp) at 71-1118 region (Figures-1-4). This primer was suitable for RABV isolate originally from Indonesia. This primer was also suitable with the isolates from Korea [11]. The samples were subjected to sequencing to obtain nucleotide sequences of N gene fragments, and homology analysis was performed to determine the identity level of Indonesia RABV isolates against Pasteur Virus (PV).

### Homology and phylogenetic analyses of Indonesian rabies virus isolates

According to the homology measurement, isolated samples had homology score of 98-99% compared to Indonesian RABV isolate submitted in GenBank (Table-2). This indicates that the virus isolated in this study is appropriate or is the same isolate as RABV from Indonesia in GenBank. The viral homology score compares with isolates from China, Thailand, India, Korea and Pasteur were 89%, 86-88%, 85-88%, and 84-85% respectively (Table-2).

**Table-2:** Homology of the isolated RABV compared to the Indonesian isolate submitted in GenBank.

Sample code	Sample origin	Indonesian RABV in GenBank (%)	Chinese RABV in GenBank (%)	Thailand RABV in GenBank (%)	Indian RABV in GenBank (%)	Korean RABV in GenBank (%)	Rabies PV in GenBank (%)
6	Kalimantan	99	92	89	88	87	85
7	Kalimantan	98	92	89	88	87	85
8	Kalimantan	99	92	89	87	87	85
C4	Sulawesi	99	92	89	87	86	85
C9	Sulawesi	98	92	89	87	87	85
148	Bali	99	92	89	-	-	-
285	Bali	99	92	88	87	85	84
382	Bali	99	92	88	86	85	84
391	Sumatera	99	93	89	87	88	85
436	Sumatera	99	93	89	87	87	85
533	Sumatera	99	93	89	87	87	85

RABV=Rabies virus, PV=Pasteur Virus

Although RABV belongs to one genotype, the homology score of isolated samples compared to RABV within Asian countries remained different. It commonly occurs in RNA virus, according to its rapid evolution. During RNA virus replication, mutation usually occurs due to lack of proofreading activity of its polymerase protein. This condition also caused by environment and host transmission. The change of both conditions are capable to trigger mutation that lead minor genomic population of RABV to become predominant. This condition makes previously and recently isolated RABV viruses are related to each other and build quasispecies[12].

**Figure-5:**
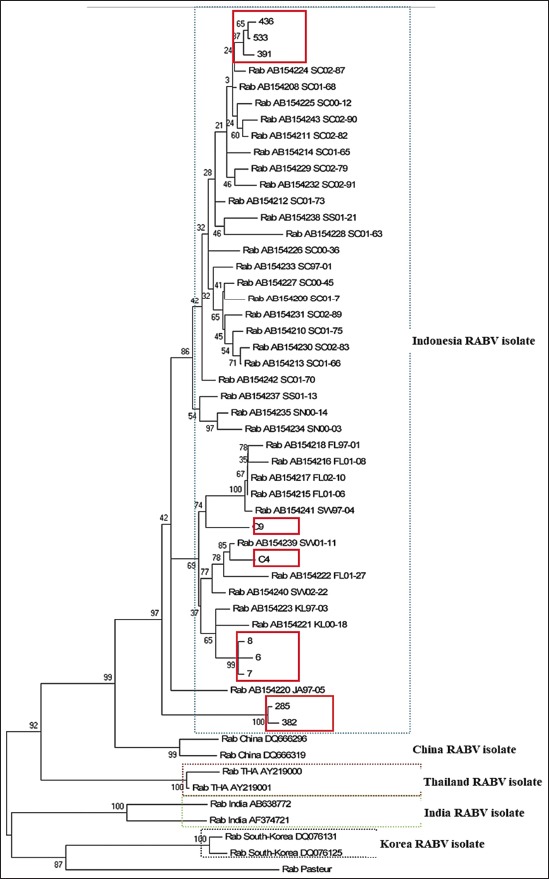
Phylogenetic tree of isolated rabies virus compared to Indonesia, China, Thailand, India, Korea, and Pasteur. Isolated samples were marked in red square.

Lack of similarity between isolated samples compared to PV caused synthetized antibody in vaccinated host against RABV is not capable to neutralize infecting RABV perfectly. It is needed to use local isolate as seed vaccine to control RABV spreading in Indonesia. In this research, homology of the samples that are isolated from Bali (148) could not be detected. The result of homology score was confirmed by phylogenetic analysis (Figure-5).

According to phylogenetic analysis, isolated samples did not share the same lineage toward PV. They had the closest relation to Chinese RABV isolate. It is proved by Zhang *et al*. [13]. The reason of close relation between RABV from China and Indonesia is unrevealed yet. It might be due to human migration from China to Indonesia. The same condition occurred in spreading of rabies disease in Indonesia. The introduction of rabies disease in free area is caused by human and animal (predominantly dog) mobility interisland [14]. The phylogenetic tree showed that the samples isolated from Sumatera shared close lineage to RABV that is previously isolated from the same island. This condition also occurred toward other isolated samples. C4 and C9 also shared the same lineage toward samples that were previously isolated from Sulawesi; the samples coded as 6, 7, and 8 shared the same lineage toward previously isolated from Kalimantan. The samples coded as 285 and 382 that were isolated from Bali built different groups compared to other isolated samples. It needs further research to understand this condition.

### Analysis of antigenic site

Antigenic site of nucleoprotein encoding gene from the isolated samples was analyzed compared to Pasteur. It was noted that mutation only occurred in antigenic Site IV (Table-3).

**Table-3:** Antigenic site of nucleoprotein encoding gene from the isolated samples compared to Pasteur.

Sample code	Sample origin	Antigenic Site IV Protein N	

		Amino acid number 247	Mutation
Pasteur		-	
6	Kalimantan	-	
7	Kalimantan	-	
8	Kalimantan	-	
C4	Sulawesi	+	(F—C) Phe – Cys
C9	Sulawesi	-	
148	Bali	-	
285	Bali	-	
382	Bali	-	
391	Sumatera	-	
436	Sumatera	-	
533	Sumatera	-	

This research showed that mutation occurred in amino acid number 247 of nucleoprotein encoding gene. The mutation from phenylalanine to cysteine occurred between samples marked C4 compared to Pasteur. It was suggested that region of antigenic site of nucleoprotein of RABV was conserved compared to other regions of nucleoprotein. Although the mutation only occurs between samples marked C4 compared to Pasteur, the homology score of isolated samples compared to Pasteur remains low.

The result of N gene homology showed that the percentage of rabies in Indonesia still has a high homology level with the previous Indonesian isolates. However, these results were different when compared to rabies strains from China, Korea, Thailand, India, and Pasteur. This study result suggests that Indonesian isolates belonged to a group with Chinese isolates. Between the isolates of Indonesia and China, the homology sequence of nucleotides reached 95%, while, for isolates from Thailand, the homology level reached only 90%. Based on the phylogenetic tree, it was found that isolated samples were separated from PV. It might be suggested that circulated RABV in Indonesia evolved due to environmental conditions that make them to have low homology level compared to PV [15]. Based on this finding, it is needed to use local strain as seed vaccine to control RABV spreading.

N gene has an important role in host adaptation pattern. It consists of 1350 bp nucleotides in length and encodes 450 amino acids. The N gene consists of antigenic Sites I and III, which play a role in the encapsidation of the genome RNA and the formation of an active cytoplasmic ribonuclease complex which is essential for viral replication. The amino acid at position (aa) 389 is the site of phosphorylation, linear to epitope 231 with tryptophan residues. There are four antigen sites in N protein. They are antigen I, II, III, and IV. Antigen I and IV are linear epitope [9].

It was noted that amino acid number 247 in antigenic Site IV region has mutated from phenylalanine to Cytosine. The previous study proved that the N gene is relatively stable against the changes, but evolution can occur based on the range of time and geographical area. Differences in the genome nucleotide cycle between viral rabies strains and vaccine strains can reach 10% [6].

Some new findings were obtained from the conducted research, such as some changes both nucleotides and amino acid from isolated samples compared to referred virus. From the N gene, the change occurs in the order of the amino acid number 247 that belongs to antigenic site IV region.

## Conclusion

The N gene fragment of RABV can be amplified using a specific primer that produces an amplicon length of 1047 bp. Isolated samples were the same species as previous RABV in Indonesia obtained from GenBank. Their homology score was 98-99%. Isolated samples had low homology score compared to Pasteur (84-85%). It was suggested that circulated RABV in Indonesia was different species compared to PV. Region of antigenic site of nucleoprotein encoding gene was conserved. Only one mutation was found.

## Authors’ Contributions

JR and SS carried out the main research works; JR and WMY analyzed the main data in the experiments; and JR, SS, WMY, and FAR approved the final manuscript. All authors read and approved the final manuscript.

## References

[1] Tang Q, Li H (2005). Epidemic situation and related factors analysis of rabies in China. Chin. J. Epidemiol.

[2] Tao X, Tang Q, Li H, Mo Z, Zhang H, Wang D, Zhang Q, Song M, Velasco-Villa A, Wu X, Rupprecht C.E, Liang G (2009). Molecular epidemiology of rabies in Southern people's republic of China. Emerg. Infect. Dis.

[3] World Health Organization (2013). WHO Expert Consultation on Rabies (Second Report).

[4] Hampson K, Coudeville L, Lembo T, Sambo M, Kieffer A, Attlan M, Barrat J, Blanton J.D, Briggs D.J, Cleaveland S (2015). Estimating the global burden of endemic canine rabies. PLoS Negl. Trop. Dis.

[5] Wunner W.H, Jackson A.C, Wunner W.H (2002). Rabies virus. Rabies.

[6] Nagaraja T, Madhusudana S, Desai A (2018). Molecular characterization of the full-length genome of a rabies virus isolate from India. Virus Genes.

[7] Hideo G, Nobuyuki M, Hiroshi I, Naoto I, Makoto S, Toshio K, Akihiko K (2000). Mapping of epitopes and structural analysis of antigenic site in the nucleoprotein of rabies virus. J. Gen. Virol.

[8] Suwarno S (2005). Molecular Characterization of Proteins and Genes the coding of Nucleoprotein and Glycoprotein Virus Rabies from Several Geographic Areas in Indonesia. Dissertation. Doctoral Program of Medical Science.

[9] Sambrook J.F, Russel D.W (2001). Molecular Cloning:A Laboratory Manual.

[10] Zhou M, Zhou Z, Kia G.S, Gnanadurai C.W, Leyson C.M, Umoh J.U, Kwaga J.P, Kazeem H.M, Fu Z.F (2013). Complete genome sequence of a street rabies virus isolated from a dog in Nigeria. Genome Announc.

[11] Yang D.K, Park Y.N, Hong G.S, Kang H.K, Oh Y.I, Cho D.S, Song J.Y (2011). Molecular characterization of Korean rabies virus isolates. J. Vet. Sci.

[12] Knipe D.M, Howley P.M (2013). Fields Virology.

[13] Zhang Y.Z, Xiong C.L, Zou Y, Wang D.M, Jiang R.J, Xiao Q.Y, Hao Z.Y, Zhang Z.L, Yu Y.X, Fu Z.F (2006). Molecular characterization of rabies virus isolates in China during 2004. Virus Res.

[14] Dibia I.N, Sumiarto B, Susetya H, Putra A.A.G, Scott-Orr H, Mahardika G.N (2015). Phylogeography of the current rabies viruses in Indonesia. J. Vet. Sci.

[15] Zhang G, Zhen F.F (2012). Complete genome sequence of a street rabies virus from Mexico. J. Virol.

